# Ethosomes: A novel drug carrier

**DOI:** 10.1016/j.amsu.2022.104595

**Published:** 2022-09-08

**Authors:** Neha Chauhan, Parul Vasava, Sharuk L. Khan, Falak A. Siddiqui, Fahadul Islam, Hitesh Chopra, Talha Bin Emran

**Affiliations:** Laxminarayan Dev College of Pharmacy, Gujarat Technological University, Bharuch, Gujarat, India; MUP's College of Pharmacy (B Pharm), Degaon, Risod, Washim, Maharashtra, 444504, India; Department of Pharmacy, Faculty of Allied Health Sciences, Daffodil International University, Dhaka, 1207, Bangladesh; Chitkara College of Pharmacy, Chitkara University, Punjab, India; Department of Pharmacy, Faculty of Allied Health Sciences, Daffodil International University, Dhaka, 1207, Bangladesh; Department of Pharmacy, BGC Trust University Bangladesh, Chittagong, 4381, Bangladesh

**Keywords:** Ethosomes, Transdermal delivery, Applications, Proteins and peptides delivery

## Abstract

Ethosomal systems are newer lipid vesicular carriers that have been around for 20 years, but over that period they have grown significantly as a means of transdermal drug delivery. They have a sizable amount of ethanol in them. These nanocarriers carry medicinal substances with various physicochemical qualities throughout the skin and deep skin layers. Since they were created in 1996, ethosomes have undergone substantial investigation; new substances have been added to their original composition, creating new varieties of ethosomal systems. These innovative carriers, which can be added to gels, patches, and lotions, are prepared using several novel methods. In addition to clinical trials, many in vivo models are employed to assess the effectiveness of dermal/transdermal administration. This review focuses on different generation of ethosomes and their comparison with other conventional liposomes.

## Highlights

• In early December 2019, a large pneumonia epidemic occurred in Wuhan, China.
• COVID-19 therapy focuses on treating the disease's symptoms.
• Globally, numerous studies and researchers have recently started fighting this virus.
• This paper examines the main characteristics of coronavirus and the computational analyses necessary to avoid infection.

## 1 Introduction

The skin is the most extensive and adaptable route for systemic and topical medication administration. The stratum corneum, the skin's outermost layer, serves as the skin's most durable barrier against drug penetration, limiting medication bioavailability when applied topically. Therefore, it is crucial to research and contrast the various carriers needed for systemic medication delivery in order to circumvent the natural skin barrier. Transdermal drug delivery is a less invasive approach to medication administration that provides controlled drug distribution, less frequent dosing, patient compliance, and prevention of first pass metabolism [[1], [2], [3]].

The development of liposomes heralded a new era in drug delivery research, and a variety of vesicular systems have subsequently been created [4]. Cevc and Blume also discovered transferosomes, which are malleable or elastic liposomes, in 1992. Transferosomes were then followed by the ground-breaking work of Touitou et al. which resulted in the discovery of a unique lipid vesicular system known as ethosomes [5].

The development of modified versions of liposomes was prompted by their lower size, lower entrapment efficiency, and negative zeta potential. Novel modified lipid carriers called ethosomes are made of ethanol, phospholipids, and water. In addition to phospholipids and water, which have been suggested to have improved vesicular properties and skin penetration, ethanol is present in quite high amounts in ethosomes [6]. Ethosomes, are again divided into binary, classical, and transethosomes based on their contents like alcohol, have quickly emerged as an unique drug delivery system [[7], [8], [9], [10]].

## 2 Ethosomal system types

**Fig. 1:**
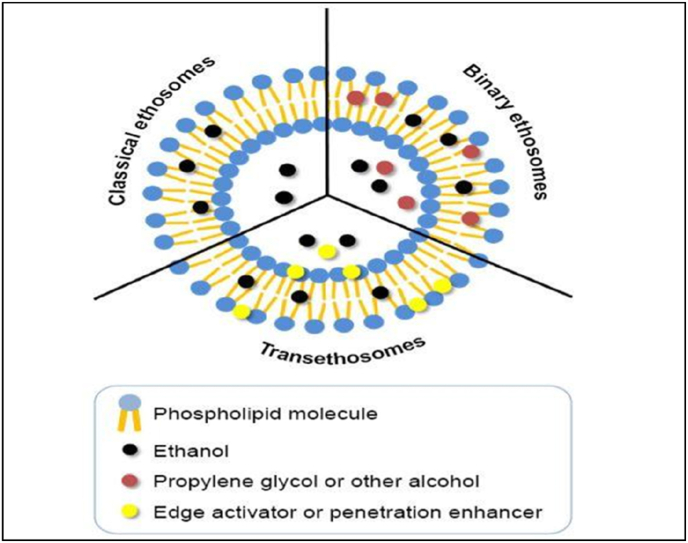
Ethosomal subtypes [11].

Ethosomes can be subdivided based on their compositions (Fig. 1). The major differences between various ethosomes for transdermal drug delivery is shown in Table 1.

**Table 1:** Difference between various ethosomes for transdermal drug delivery [11].

Sl. No.	Parameter	Classical ethosomes	Binary ethosomes	Transethosomes
1	Composition	1. Phospholipids	1. Phospholipids	1. Phospholipids
		2. Ethanol	2. Ethanol	2. Ethanol
		3. Stabilizer	3. Propylene glycol (PG) or other alcohol	3. Edge activator (surfactant) or penetration enhancer
		4. Charge inducer	4. Charge inducer	4. Charge inducer
		5. Water	5. Water	5. Water
		6. Drug/agent	6. Drug/agent	6. Drug/agent
2	Morphology	Spherical	Spherical	Regular or irregular spherical shapes
3	Size	Smaller than the classical liposomes	Equal to or smaller than classical ethosomes	Size based on type and concentration of penetration enhancer or edge activator used
4	Entrapment efficiency	Superior than traditional liposomes	Often higher than traditional ethosomes	Higher than the majority of typical ethosomes
5	Skin permeation	Usually greater than traditional liposomes	Usually on par with or superior to traditional ethosomes	Often higher than traditional ethosomes
6	Stability	More robust than traditional liposomes	Stabler than classical ethosomes	There was no clear trend found

## 3 HOW do ethosomes work?

Vesicles, ethanol, and skin lipids interact synergistically in ethosome function [11]. Because ethosomes and skin lipids interact better than liposomes, they improve the distribution of active ingredients over liposomes. When ethanol interacts with the lipid molecules in the polar head group region, the transition temperature of the lipids in the stratum corneum is decreased. These cause the drug to be delivered into the deep layers of the skin by increasing fluidity and lowering lipid multilayer density. Furthermore, ethanol imparts smoothness and flexibility to vesicles, facilitating deeper penetration into the epidermal layer [12].

## 4 Preparation of ethosomes

The hot approach and the cold method are two often employed techniques needed to create ethosomes.

### 4.1 Cold method

At room temperature, with vigorous shaking, the ethanol is dissolved in the phospholipids, medicine, and other lipid components. The jar is then heated to 30 °C. This is widely used and is referred to as the “cold approach. In another beaker, water is heated to 30 °C before being introduced and continuously swirled into the initial mixture. Vesicles start to emerge after 5 min of churning. It's important to keep produced vesicles cold [13].

### 4.2 Hot method

The hot procedure entails combining the medication with ethanol and propylene glycol. At 40 °C, phospholipid dispersion in water is created. This dispersion is combined with a previously produced mixture. Size reduction is next accomplished by sonication or extrusion after this final combination is heated to 30 °C [13].

## 5 Characterization

The production of the ethosomal vesicles can be evaluated using photomicrographs, transmission electron microscopy (TEM), and scanning electron microscopy (SEM) micrographs [14]. The formulation's zeta potential can be determined using a zeta metre [15]. The reduction in mean vesicle diameter can vary depending on the ethanol and phospholipid concentrations [[16], [17], [18]]. The transition temperature of the vesicular lipid systems can be measured using differential scanning calorimetry, a method that can be used to detect ethanol-skin phospholipid interaction, a property connected to the fluidizing impact of ethanol on the phospholipid bilayers [19].

The ultracentrifugation method can be used to determine the ethosomes' degree of entrapment. The high level of lamellarity and the presence of ethanol in the vesicles can be used to explain why ethosomes can effectively entrap hydrophilic and lipophilic medicines. Additionally, ethosomal formulations have a better capacity for trapping than liposomes [20].

A1 Encapsulation efficiency = (A1 – A2) *100/

A1: Initial dosage; A2: Initial dosage as measured by spectrophotometry in the filtrate; A1-A2 shows how much of the medicine is contained in the formulation. The ability of the ethosomal preparation to penetrate the epidermal layers can be evaluated using confocal laser scanning microscopy. Studies on the capacity of the ethosomal formulation to increase the penetration of both hydrophobic and hydrophilic molecules through the skin in both in vitro and in vivo settings have shown that it is superior to standard liposomes [21,22].

Why ethosomes for transdermal drug delivery?

Although there are other vesicular carriers for transdermal drug administration, including liposomes, niosomes, and transferosomes, ethosomes have grown to be highly recommended for a variety of reasons. Traditional liposomes do not deeply penetrate the stratum corneum's top layer, making them of little to no value as transdermal delivery vehicles. Niosomes, non-ionic surfactant vesicles that may encapsulate a wide spectrum of solutes, are less effective in penetrating skin than ethosomes. Similar to ultra-deformable or elastic carriers, transferosomes have a high penetration power due to their great deformability but are unable to penetrate deeper skin layers. For systemic effects, it is therefore less effective [23]. Numerous vesicular carriers, such as liposomes, niosomes, and transferosomes, are accessible for the transdermal administration of drugs. Ethosomal carriers, on the other hand, are systems that comprise soft vesicles and are primarily made of water, ethanol at a relatively high concentration, and phospholipid (Phosphatidyl choline). They pierce the skin, enabling improved distribution of different chemicals to the skin's deep layers or to the circulatory system. Both ethosomal lipids and the mortar's bilayers are fluidized by the ethanol in the ethosomes. The lipid bilayers are then broken down by the soft, pliable vesicles. As a result, they have lately become a cutting-edge medication delivery technology for transdermal distribution [6].

## 6 Ethosomal system-based marketed products

Professor Elka Touitou, a research development company (Yissum) from the Hebrew University of Jerusalem, and Trima, a pharmaceutical business, collaborated to create an ethosomal cream containing acyclovir (Supra-vir). Herpes simplex virus infections of the skin and mucous membranes were treated with the preparation [24]. The website of the producing company, however, no longer has access to the product information. Some authors have also mentioned Nanominox, Cellutight EF, Skin Genuity, and Decorin cream as other ethosomal system-based goods [25]. Although these products were made a few years ago, there is currently no comprehensive information online about them or the companies that made them.

## 7 Applications

• Ethosomes have been shown in numerous trials to be an effective treatment for viral and microbial skin infections. Animal models of deep skin infections were used to create and test the efficacy of the bacitracin and erythromycin ethosomal systems [26].
• When manufactured, ammonium glycyrrhizinate ethosomes were shown to have an anti-inflammatory impact on the skin of human volunteer subjects.
• When tested in vivo on rabbits, ethosomal patches in treating androgen insufficiency in males and menopausal symptoms in women have sufficiently demonstrated better results.
• Research suggests that ethosomes may have analgesic, antipyretic, and efficacious effects against erectile dysfunction.
• Research has also indicated that ethosomes might be utilised to topically transport DNA molecules for skin cells to express certain genes.

## 8 Advantages of ethosomal drug delivery [27,28]

• With this delivery technology, proteins and peptides can be easily supplied [27,28].
• The raw materials are harmless and readily penetrate the skin.
• This delivery technology is not just useful for pharmaceuticals; it may also be used in other industries, such as veterinary medicine and cosmetics.
• Patient adherence with the ethosomal drug delivery technology is quite high. Because the ethosomal medication is administered in gel or cream in semisolid form for high patient compliance.
• A straightforward drug delivery approach that can be used to contrast complex methods like phonophoresis and iontophoresis.
• The Ethosomal system may be immediately commercialised and is passive and non-invasive.

## 9 Limitations of ethosomal drug delivery

• Increased levels are needed. Only powerful compounds with a daily intake of 10 mg or less are allowed [27,28].
• Instead of offering moderate, continuous medication delivery, it is often intended as a method of achieving quick bolus type drug input.
• Sufficient solubility of the medicine to penetrate cutaneous microcirculation and enter the systemic circulation in both lipophilic and watery conditions.
• The drug's molecular size needs to be appropriate for percutaneous absorption.
• Not all varieties of skin will adhere to adhesive as well.
• It might not be cost-effective.
• A low yield.
• Dermatitis or skin irritation brought on by excipients and enhancers used in medication delivery systems

## 10 Conclusion

Ethosomes have been proven as an emerging and effective carrier system over two decades. Their ability to provide effective therapeutic effect, topically and systemically through skin have made them an appealing and novel carrier system over time. Profound investigation has also resulted in the development of a new generation of ethosomal systems known as Transethosomes. Over classical ethosomes these transethosomes have better skin permeation and systemic circulation. Continuous research has been carried on ethosomal systems and research supports that these carriers along with penetration enhancers could be proven effective as vehicles for delivering drug in form of gels, patches and creams. More detailed studies however are needed to further work on the stability of the ethosomal system.

## FUNDING

No funding.

## Data availability statement

The data that support the findings of this study are available from the corresponding author upon reasonable request.

## Ethical approval

This article does not require any human/animal subjects to acquire such approval.

## Please state any sources of funding for your research

This study received no specific grant from any funding agency in the public, commercial, or not-for-profit sectors.

## Author contribution

**Neha Chauhan:** Conceptualization, Data curation, Writing-Original draft preparation, Writing- Reviewing and Editing. **Parul Vasava:** Conceptualization, Writing-Reviewing and Editing, Visualization. **Sharuk L. Khan, Falak A. Siddiqui:** Data curation, Writing-Original draft preparation, Writing- Reviewing and Editing. **Fahadul Islam, Hitesh Chopra:** Data curation, Writing-Original draft preparation, Writing- Reviewing and Editing. **Talha Bin Emran:** Conceptualization, Writing-Reviewing and Editing, Visualization.

## Please state any conflicts of interest

All authors report no conflicts of interest relevant to this article.

## Registration of research studies

1. Name of the registry: Not applicable.
2. Unique Identifying number or registration ID:Not applicable.
3. Hyperlink to your specific registration (must be publicly accessible and will be checked): Not applicable.

## Guarantor

Talha Bin Emran, Ph.D., Associate Professor, Department of Pharmacy, BGC Trust University Bangladesh, Chittagong 4381, Bangladesh. T: +88-030-3356193, Fax: +88-031-2550224, Cell: +88–01819942214. https://orcid.org/0000-0003-3188-2272. E-mail: talhabmb@bgctub.ac.bd.

## Consent

Not applicable.

## Declaration of competing interest

The authors declare that they have no conflicts of interest.
